# A Pilot Investigation Into the Use of Teledentistry and Artificial Intelligence to Assess Dental Erosion in Competitive Swimmers

**DOI:** 10.1002/cre2.70018

**Published:** 2024-11-07

**Authors:** Jacopo Lanzetti, Federica Ferrati, Lorenzo Pavone, Federico Mussano

**Affiliations:** ^1^ Department of Oral Rehabilitation and Maxillofacial Prosthesi—Dental School University of Turin Turin Italy; ^2^ Bone and Dental Bioengineering Laboratory, Department of Surgical Sciences, CIR Dental School University of Turin Turin Italy

**Keywords:** artificial intelligence, competitive swimmers, dental erosion, sport medicine, teledentistry

## Abstract

**Objective:**

The aim of the study was to assess the prevalence of dental erosion in competitive swimmers using teledentistry and artificial intelligence.

**Materials and Methods:**

An opportunistic sample of 20 competitive swimmers was recruited. The participants reported that they carried out an average of 2.40 h of training per day, 4.45 days per week. Data gathering was carried out remotely. The subjects completed a digital questionnaire and uploaded three photos of their mouth. Intraoral photographs were analyzed using the “Intact‐Tooth” application to assess dental erosion. A statistical analysis was carried out to verify a possible correlation between the collected data.

**Results:**

The average calculated Basic Erosive Wear Examination (BEWE) index was 13.95, and 11 subjects (55%) had a severe BEWE (≥ 14). More than 40% of the participants disagreed about having social issues related to tooth hypersensitivity. Considering only subjects with a severe total BEWE value, we have observed an indirect correlation between the degree of dental erosion and diet (Pearson coefficient *r* = −0.57), whereas a direct correlation was observed between dental erosion and age (*r* = 0.493) and between BEWE and weekly training hours (*r* = 0.217).

**Conclusions:**

Because of their lifestyle, competitive swimmers can be considered at a higher risk of developing dental erosion. In this context, teledentistry and AI tools can be effectively used to intercept those at the highest risk and prevent the occurrence of conditions.

**Summary:**

Dental erosion assessment in competitive swimmers using teledentistry and AI.

## 1 Introduction

Dental erosion is a multifactorial condition, generated by the interaction of chemical, biological, and behavioral factors, that consists in the progressive loss of the dental surface, manifesting itself mainly as a result of a chemical process (Hans et al. 2016; Lussi and Jaeggi 2008).

The factors responsible for prolonged exposure to acidic substances can be intrinsic, such as gastric acid related to gastroesophageal reflux disease and eating and/or psychological disorders, or extrinsic, including the intake of acidic food substances and/or exposure to chlorine, citric acid, acetic acid, or carbonic acid (Lussi and Jaeggi 2008; Wiegand and Attin 2007).

One of the consequences of erosion is dentinal hypersensitivity, a transient, intermittent, acute pain due to dentin exposure that could be triggered by thermal, tactile, chemical, or osmotic impulses. Once exerted on the area of exposed dentin, these stimuli could induce pain without changes in the dentin–pulp complex (Berman 1985). An acidic diet and the presence of bacterial plaque on root surfaces have a demineralizing effect on these areas and can be considered risk factors for dental erosion (Hans et al. 2016; Lussi and Jaeggi 2008). Furthermore, some professions are more exposed to dental erosion than others (Wiegand and Attin 2007). Those who are employed in battery, fertilizer, and ammunition production plants are more exposed to industrial acids in the air. Some sports categories can also be considered at higher risk, such as swimmers, due to prolonged contact of dental elements in chlorinated pool water (Donovan et al. 2021).

The main causes of dental erosion in competitive swimmers are represented by oral breathing during workouts in water, which causes the dehydration of the oral cavity and a reduction in salivary flow, and by the chlorinated environment, or by chlorinated water and air breathed inside the pool. The susceptibility to dental erosion resulting in hypersensitivity is directly proportional to the time of exposure to the chlorinated environment, and then to the hours of training.

According to the WHO guidelines (World Health Organization 2006), the pH of the pool water must be between 7.0 and 7.4. The recommended value is 7.2 so that chlorine has maximum antibacterial effectiveness while remaining safe for eyes and mucous membranes.

Chlorine in pool water tends to combine with other substances such as chloramines, which evaporate in the form of sodium hypochlorite making the external environment and the air breathed more acidic (Buczkowska‐Radlińska et al. 2013).

Chloramines are highly irritating to the skin, mucous membranes (e.g., oral mucosa), and eyes. In addition, they can form when chlorine comes into contact with sweat, hair, or urine (World Health Organization 2006).

In addition, the competitive swimmer diet could also be considered a risk factor for the development of erosions because athletes during their sports or competitions often take energy drinks with a high erosive potential (Hans et al. 2016).

The available epidemiological surveys performed on the prevalence of dental erosion in swimmers, compared to subjects who do not practice this sport, report that 3% of nonswimmers, 12% of swimmers, and 39% of swim team members suffered from dental erosion (Centerwall et al. 1986), supporting the hypothesis that there could be a correlation between the outbreak of dental erosion and the composition and pH of the pool water, due to incorrect monitoring or insufficient water buffer system (Gabai et al. 1988).

Another study (Buczkowska‐Radlińska et al. 2013) analyzed the prevalence of erosion in two different groups of swimmers: competitive and noncompetitive. Dental erosion was found to be present in more than 26% of competitive swimmers and 10% of noncompetitive swimmers. Moreover, in noncompetitive swimmers, the lesions occurred exclusively on the palatal–lingual surfaces of the anterior teeth, whereas in the agonist swimmers, they affected both the vestibular and the palatal–lingual surfaces of the anterior teeth. In both categories, dental erosion involved two or more teeth, more frequently on the vestibular surfaces of the central incisors. A previous study (Rao et al. 2019) found that in a sample of 56 competitive swimmers, 48.2% had dental erosion and the prevalence of dental erosion increased proportionally, from 26% to 69%, along with the duration of years of sporting activity.

Early detection of dental erosion can be challenging, but it is crucial to implement prevention and preserve dental tissue. Lack of diagnostic devices for the assessment of dental erosion leads to difficulties in early diagnosis (Joshi et al. 2016).

To intercept dental erosions and manage them properly, it is important to carry out a careful clinical and medical assessment to identify risk factors. The diagnosis of erosive processes could be difficult, but it is important to do it at an early stage because the erosion's progression is asymptomatic, especially during the initial phase of demineralization.

In this regard, different teledentistry tools could provide a valid and effective method to early intercept dental erosions.

Especially in specific situations, teledentistry has facilitated traditional face‐to‐face dentist–patient interaction, providing an effective substitute for online consultation, exchange of investigations, and treatment planning (Maqsood et al. 2021). Online consultations with an oral health expert could minimize costs, maximize time, and provide more affordable care options for both patients and professionals (Islam et al. 2022). A previous project (Inquimbert et al. 2023) showed that teledentistry allows remote screening and consultation sessions in subjects with special needs or risks, such as competitive swimmers. In this context, teledentistry is a central tool for early diagnosis because the clinician can intercept the lesion before the patient feels the symptom and goes to the office. This way, the professional can prevent acute symptoms and the extent of the injury.

To assess the prevalence of dental erosion, different epidemiological studies employ various methodologies and different indices (Joshi et al. 2016). In vivo assessments are the gold standard for epidemiological studies and have the advantage of evaluating erosion directly in the patient's mouth. Techniques for in vivo assessment of erosion include the use of photographs and indexes (Joshi et al. 2016). In epidemiology, photographs are useful for measuring enamel defects and dental erosion as they give results similar to visual examination but may underestimate the extent of lesions (Joshi et al. 2016).

Nowadays, in addition to teledentistry, artificial intelligence (AI) could be used in medicine and dentistry with many applications dedicated to radiographic or photographic diagnosis (Müller et al. 2021; Prados‐Privado et al. 2020). In oral hygiene, as in all branches of dentistry, the use of AI is increasing rapidly (Tay et al. 2023). AI can also be used in the planning of more effective therapies, prevention, and reduction of treatment costs (Mupparapu, Wu, and Chen 2018; Hamet and Tremblay 2017). The advantages of using AI are better efficiency and precision, better patient monitoring, and time optimization (Ossowska, Kusiak, and Świetlik 2022).

The objective of this study was to assess the prevalence of dental erosion in competitive swimmers using teledentistry and AI. A possible correlation between the length of time in the chlorinated environment, the diet of the athletes, and the presence of dental erosions has been researched.

## 2 Materials and Methods

An analytical cross‐sectional design was used.

An opportunistic sample of 20 athletes of aquatic disciplines such as competitive swimming, cross‐country swimming, synchronized swimming, rescue, and water polo was selected. Rescue swimmers were included in the study because, although they are not competitive swimmers, their training involves many hours in chlorinated water per day for several days a week.

The entire sample was recruited among athletes over 18 years from sports facilities in Piedmont and Umbria, Italy, who have agreed to participate in the study voluntarily and anonymously.

The research received a favorable opinion from the Turin Dental School's Internal Ethics Committee, and all participants were informed about the study and signed an informed consent.

Data gathering was carried out remotely, from April 2023 to June 2023. All athletes completed a digital questionnaire in Google Forms, collecting data on:

- registry;
- medical history (being particularly careful with all diseases related to gastroesophageal reflux or recurrent episodes of emesis);
- eating habits (consumption of acidic foods and beverages);
- training regimen (water discipline, daily training hours, and weekly training days);
- dentinal sensitivity detected via VAS scale (detecting hot, cold, and sweet impulses) (Rocha et al. 2020);
- the Dentine Hypersensitivity Experience Questionnaire (DHEQ; Machuca et al. 2014).

The evaluation of dietary habits by questionnaire focused on the frequency of daily intake of acidic fruits, acidic drinks, sweets, and foods containing vinegar. In addition, it was investigated whether the consumption of these foods took place by leaving them in contact with the teeth for a long time (e.g., slow chewing or sipping). A high risk of diet‐related erosion was assessed if the patient consumed acidic foods more than three times a day or consumed them slowly. A medium risk was assessed if the consumption of such foods was limited to a maximum of three times a day and a low risk if the patient has this type of feeding occasionally. High‐risk patients were given a score of 4 and medium‐risk patients a score of 2.

At the end of the module, participants were asked to upload three photos of their mouth (frontal, upper occlusal, and lower occlusal) on the platform, taken with their smartphone following a previously provided tutorial.

Intraoral photographs were analyzed using the “Intact‐Tooth” application to assess the level of erosion of the enamel surface and classify the individual susceptibility (Butera et al. 2022). In addition to the immediate purpose of evaluating the photo, the app saved the analyzed images and allowed the AI machine to learn how to identify areas of erosion.

Thanks to the app, the professional highlighted the affected areas directly on the uploaded photo, and, through a precise algorithm, Basic Erosive Wear Examination (BEWE; Bartlett, Ganss, and Lussi 2008) and patient susceptibility indices were calculated.

The algorithm, based on the evaluation of almost 4000 cases of dental erosion examined by experienced dental practitioners, has calculated the score to be assigned to each area of the tooth. In any case, the operator has the possibility to change the score given by the app according to his experience, improving the accuracy of the algorithm (Butera et al. 2022).

**Figure 1:**
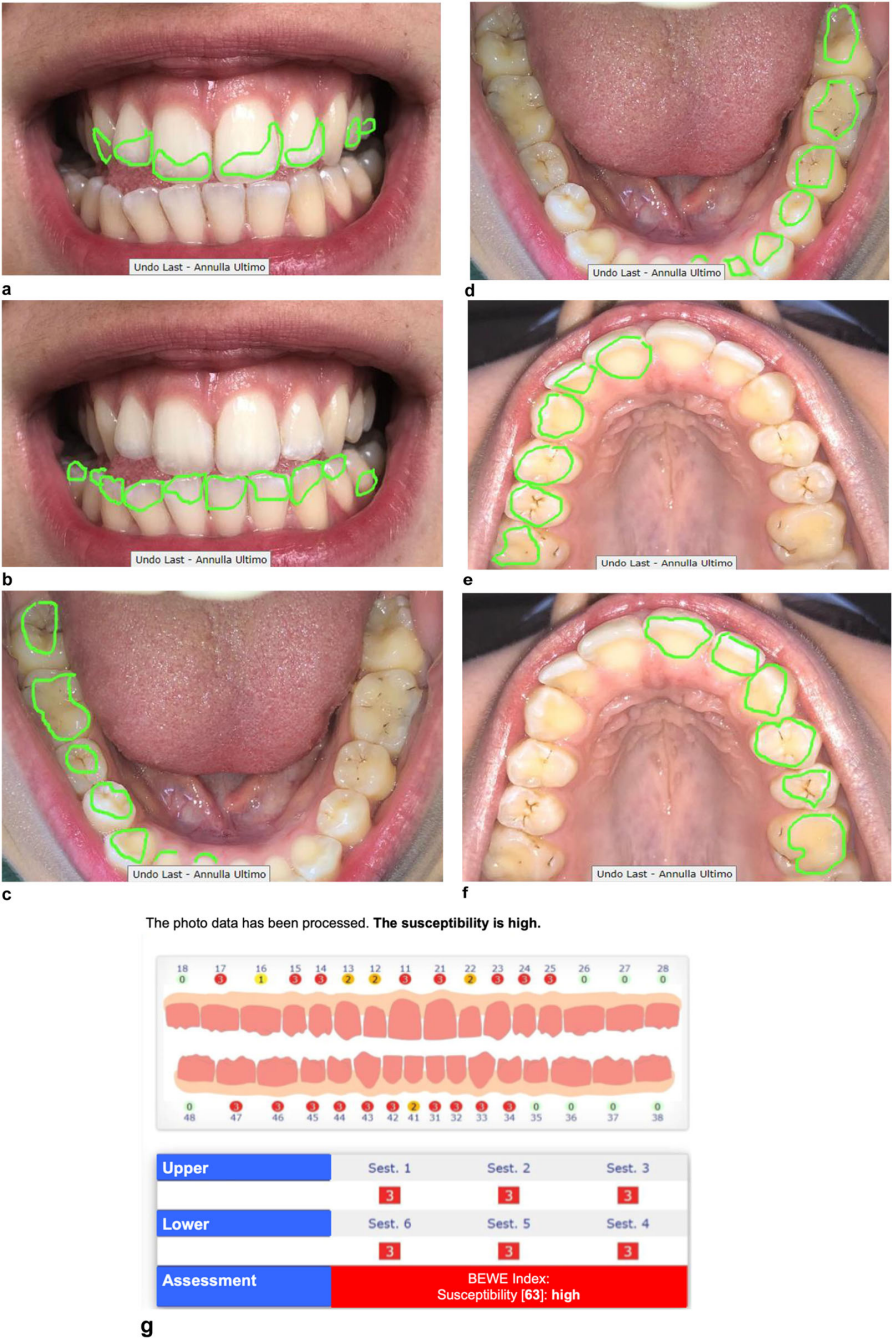
Assessment of dental erosions via the “Intact‐Tooth” app: (a) frontal, upper elements; (b) frontal, lower elements; (c) occlusal, 4° quadrant; (d) occlusal, 3° quadrant; (e) occlusal, 1° quadrant; (f) occlusal, 2° quadrant; (g) app response: BEWE index and susceptibility.

The evaluation of dental surfaces using AI was carried out by an examiner who was instructed to use the “Intact‐Tooth” app. Figure 1 shows the erosion assessment process using the intact tooth app.

The collected data were recorded in a spreadsheet of Microsoft Excel 2021 (Microsoft Corp., Redmond, WA, USA). The quantitative variables were synthesized by averages and standard deviations. A statistical analysis was performed to verify a possible correlation between the duration of the stay in the chlorinated environment, the diet of the athletes, the presence of dental erosions, and the experience and intensity of dentinal hypersensitivity by calculating the Pearson correlation index (*r*). The power analysis was not performed because the study was conducted with a small sample size.

## 3 Results

In this study, an opportunistic sample of 20 competitive swimmers (five males and 15 females) with an average age of 27–69 years was recruited.

The collected data are summarized in Tables 1 and 2.

**Table 1:** Characteristics of the sample.

Gender	Male, *n* (%)		Female, *n* (%)
	5 (25)		15 (75)
Aquatic discipline	Synchronized swimming *n* (%)	Lap swimming *n* (%)	Rescue swimming *n* (%)
	5 (25)	13 (65)	2 (10)
Erosion dietary risk	Low *n* (%)	Medium *n* (%)	High n (%)
	0 (0)	16 (80)	4 (20)
BEWE	Mild *n* (%)	Moderate *n* (%)	Severe *n* (%)
	2 (10)	7 (35)	11 (55)

**Table 2:** Average and standard deviations of the data collected.

	Average ± standard deviation
Age	26.30 ± 8.29
Hours of training per day	2.40 ± 0.88
Day of training	4.45 ± 1.61
Hours of training for week	11.60 ± 6.45
BEWE	13.95 ± 3.72
VAS	5.35 ± 4.78

The selected subjects practised different aquatic disciplines: five of them practised synchronized swimming, 13 practised pool swimming, and two practised rescue swimming. They carried out on average 2.40 h of training per day for 4.45 days per week.

pH range of chlorine in pool water was 7.4–7.6.

Four subjects (20%) were at high risk of erosion due to their diet, whereas 16 subjects (80%) were at medium risk.

The average BEWE calculated is 13.95. Eleven subjects (55%) had a severe BEWE (≥ 14), whereas the average value of perceived pain, analyzed, according to the VAS scale, is 5.35.

Table 3 shows the most frequent responses of competitive swimmers to the DHEQ‐15.

**Table 3:** Most frequent responses from the Dentinal Hypersensitivity Experience Questionnaire‐15.

Question number	Questions	*n* (%)	Response
	Restrictions		
1	Having sensations in my teeth takes a lot of the pleasure out of eating and drinking	7 (35)	Disagree
2	It takes a long time to finish some foods and drinks because of sensations in my teeth	6 (30)	Disagree
		6 (30)	Strongly Disagree
3	There have been times when I have had problems eating ice cream because of these sensations	7 (35)	Agree
	Coping		
4	I have to change the way I eat or drink certain things	8 (40)	Disagree
5	I have to be careful how I breathe on a cold day	9 (45)	Disagree
6	When eating some foods, I have made sure they do not touch certain teeth	8 (40)	Strongly disagree
7	Because of the sensations, I take longer than others to finish a meal	8 (40)	Disagree
		8 (40)	Strongly disagree
	Social		
8	I have to be careful what I eat when I am with others because of the sensations in my teeth	11 (55)	Disagree
9	Going to the dentist is hard for me because I know it is going to be painful as a result of sensations in my teeth	8 (40)	Strongly disagree
	Emotions		
10	I have been anxious that something I eat or drink might cause sensations in my teeth	9 (45)	Disagree
11	The sensations in my teeth have been irritating	10 (50)	Agree
12	The sensations in my teeth have been annoying	8 (40)	Agree
	Identity		
13	Having these sensations in my teeth makes me feel old	7 (35)	Strongly disagree
14	Having these sensations in my teeth makes me feel damaged	5 (25)	Strongly disagree
15	Having these sensations in my teeth makes me feel as though I am unhealthy	6 (30)	Strongly disagree

Among participants with dentinal hypersensitivity, seven subjects (35%) had issues eating cold foods such as ice cream, but eight subjects (40%) didn't report changes in eating patterns. More than eight participants (40%) denied having social issues related to tooth hypersensitivity; however, eight of them (40%) claimed to have emotional issues related to this condition. Finally, over five subjects (25%) did not think that these feelings were of concern for health.

Correlation between the different parameters was assessed through the Pearson correlation index, and no correlation between age and BEWE (*r* = −0.063) or age and VAS (*r* = −0.017) was found. Through the collected data, it was possible to demonstrate a direct correlation between weekly training hours and BEWE (*r* = 0.170) and an indirect correlation between training hours and VAS (*r* = −0.261). Furthermore, an indirect correlation was found by comparing acidic diet and BEWE (*r* = −0.199).

**Figure 2:**
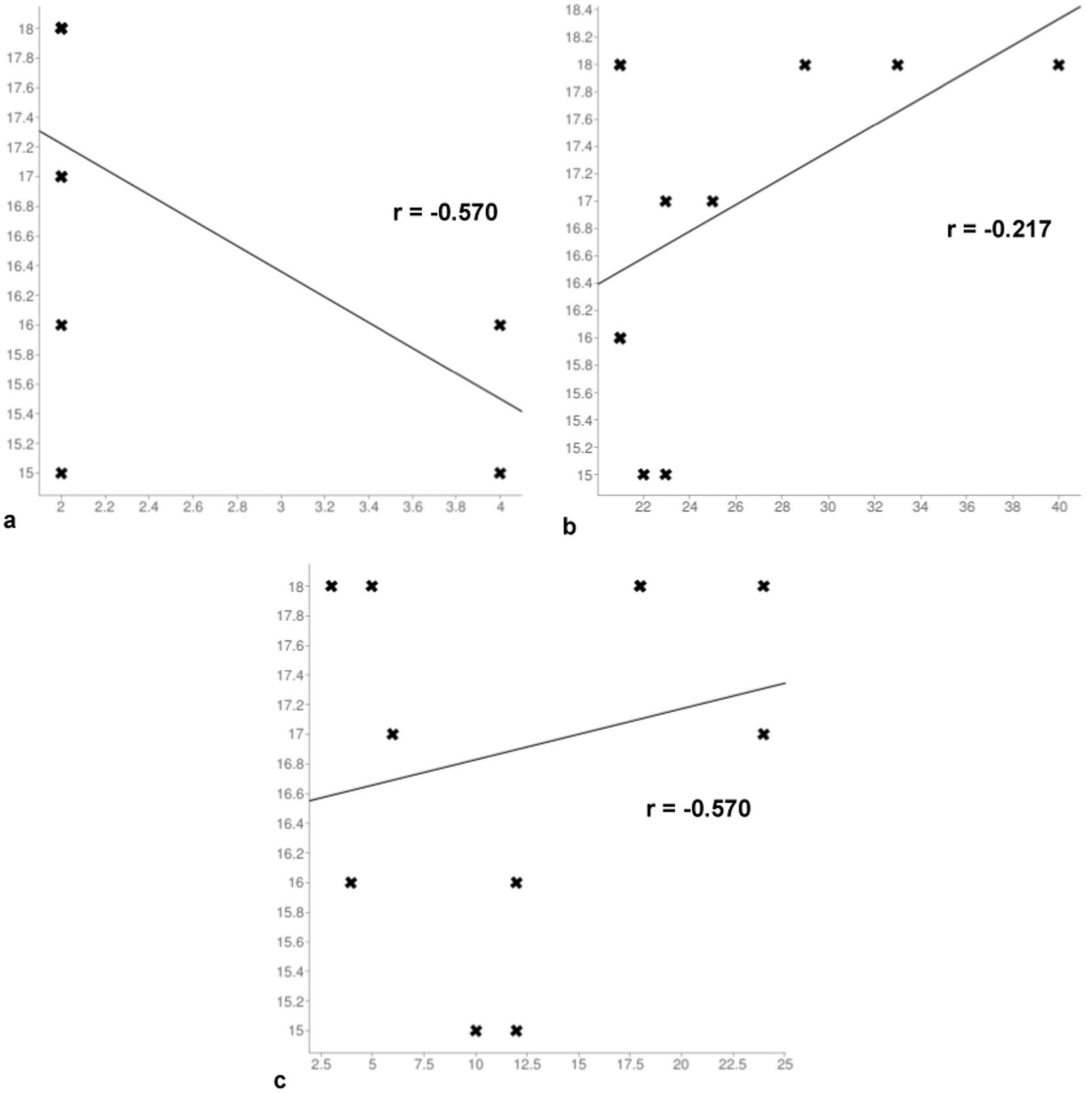
Assessment of risk factors in subjects with severe dental erosion (BEWE ≥ 14) among *x* and y values, respectively. (a) Linear regression between BEWE and diet (*r* = −0.570); (b) linear regression between BEWE and age (*r* = 0.493); (c) linear regression between BEWE and weekly training hours (*r* = 0.217).

Taking into account only subjects with a total severe BEWE value (≥ 14), an indirect correlation was found between the degree of dental erosion and diet (*r* = −0.57), whereas there was a direct correlation between dental erosion and age (*r* = 0.493) and between BEWE and weekly training hours (*r* = 0.217). Figure 2 shows the respective linear regressions.

The values of the Pearson coefficient (*r*) of correlation between all the parameters evaluated are shown in Table 4.

**Table 4:** Pearson's coefficient values (*r*) of correlation between data evaluated.

Data 1	Data 2	Pearson's coefficient (*r*)
Age	BEWE	−0.063
Age	VAS	−0.017
weekly training hours	BEWE	0.170
weekly training hours	VAS	0.261
Diet	BEWE	−0.199
Diet	VAS	0.177
VAS	BEWE	−0.150

## 4 Discussion

The aim of this study was to assess the prevalence of dental erosion in competitive swimmers through the use of teledentistry and AI.

In the present study, all the selected competitive swimmers showed dental erosion. These data are higher compared to similar studies conducted in other countries (Buczkowska‐Radlińska et al. 2013; Zebrauskas, Birskute, and Maciulskiene 2014).

The results of this study showed that athletes engaged in competitive swimming, with more hours of training in the pool water, had a high incidence of dental erosion, contrary to a prevalence of 2% of competitive swimmers who has trained in water with a pH from 7.20 to 9.0 showed by a previous study (D'Ercole et al. 2016).

Through the evaluation of the DHEQ‐15 questionnaire, it has been concluded that the presence of dental erosion associated with hypersensitivity appeared in most subjects examined for discomfort and pain. Although the level of individual susceptibility and the values of the VAS scale were high, the symptomatology did not influence negatively the lifestyle and behavior of participants, specifically, the dietary variety, the way a particular food or drink was taken (especially some types that can trigger increased hypersensitivity), or the level of confidence with their mouth.

No significant correlation has been found between the dental erosion and the swimmers' age. Regarding the relationship between the presence of erosion and the acidic diet, this study showed an indirect correlation (*r* = −0.199).

This correlation was at odds with most scientific studies (Salas et al. 2015), which could be attributed to the fact that the assessment of dietary habits proposed in our study only takes into account the daily frequency of acidic food intake and does not evaluate, the intake of other foods with greater buffering capacity, or the patient's implementation of preventive dental procedures (e.g., fluoroprophylaxis).

Pearson coefficients calculated on subjects with severe dental erosion (BEWE ≥ 14) were found to be more significant, particularly swimmers' age (*r* = 0.493) and weekly training hours (*r* = 0.217).

The results of this study suggested that there was a correlation between dental erosion and individual characteristics and the patient's lifestyle. For this reason, the prevention modalities for the onset and progression of dental erosion are based on the interception of possible initial lesions, associated with remineralizing and desensitizing home and professional treatments. This must be accompanied by a survey of the individual's lifestyle. Personalized oral hygiene treatments are also required, as well as dietary advice and optimization of fluoride treatment.

Considering erosion as a condition linked to the sporting environment, measures are needed to promote health in high‐risk individuals. Therefore, regular dental checks are recommended to identify early injuries and plan prevention strategies. In this case, teledentistry could be a valuable help (Wiegand and Attin 2007). The integration of a dental consultation through telemedicine in environments frequented by people at risk of dental diseases, as in this case of study swimming pools, could provide several advantages, such as collecting data from the patient and optimizing the dental checks in a certain population (Giraudeau et al. 2021).

Although teledentistry is an expanding area, there are still some obstacles to its use (Maqsood et al. 2021). In particular, the small sample size of our study showed that it is a practice that the patient struggles to use, especially for a first visit.

In addition to teledentistry, AI technology has influenced the healthcare field, making the diagnosis of dental pathologies more accurate.

This cross‐sectional study was conducted using teledentistry and AI. Thanks to teledentistry, it was possible to improve access to the dental examination, optimizing costs and resources. The use of the Intact‐Tooth app and AI allowed a nonpersonal assessment of dental erosions.

The Intact‐Tooth app could be defined as an assisted diagnostic tool. In the future, thanks to the implementation of the AI machine, the app will be able to provide the operator with a collective clinical experience, assuming the value of an accurate and complete diagnostic tool, that can help and not replace the operator in dental erosion early assessment.

Currently, to minimize evaluation and diagnosis errors, it is always necessary to combine assessment with AI and conventional examination. Therefore, it is necessary that the devices using AI for patient assessment leave a predominant role to the practitioner who must have the final decision on diagnosis or the application of treatment.

Also for the legal issues, the practitioner cannot delegate the acquisition of informed consent, especially in a context such as teledentistry where the patient may not fully understand the functioning of AI.

AI‐based technology has the potential to improve oral health care, but at the same time, there is a need to define clinical and forensic responsibilities, especially when AI makes errors in diagnosis (Sallam 2023). Several reviews (Tay et al. 2023; Sallam 2023) highlighted the lack of guidelines and ethical codes for the use of AI in dentistry.

It is also important to stress that some of the AI technologies used are owned by private entities, and it is therefore necessary to ensure the privacy of the patient who should be aware of how their data are being handled.

The limitations of this study include variables associated with the oral hygiene habits of each athlete, which can affect the formation of dental erosions.

In addition, it would be necessary to analyze the prevalence of dental erosion even in a control group of non‐swimmers or noncompetitive swimmers.

The sample size analyzed in this study was small and therefore did not provide sufficient evidence to determine a statistical correlation with the population as a whole. In the future, it will be important to obtain a larger sample of swimmers.

## 5 Conclusions

In this small pilot study, it was found that factors that increase the risk of dental erosion depend on the lifestyle of the athletes including the duration of swimming and the amount of training.

In this context, teledentistry and AI tools could be used effectively to intercept those at the highest risk and prevent the occurrence of conditions.

## Author Contributions

Jacopo Lanzetti conceived the idea; Jacopo Lanzetti and Federica Ferrati collected and analyzed the data and contributed to the writing; Lorenzo Pavone and Federico Mussano contributed to the writing and critically revised the manuscript.

## Conflicts of Interest

The authors declare no conflicts of interest.

## Data Availability

The data that support the findings of this study are available from the corresponding author upon reasonable request.
